# Intravitreal Ranibizumab Injection as an Adjuvant in the Treatment of Neovascular Glaucoma Accompanied by Vitreous Hemorrhage after Diabetic Vitrectomy

**DOI:** 10.1155/2016/4108490

**Published:** 2016-05-16

**Authors:** Xi Shen, Yanwei Chen, Yanuo Wang, Lu Yang, Yisheng Zhong

**Affiliations:** ^1^Department of Ophthalmology, Ruijin Hospital, School of Medicine, The Jiaotong University, Shanghai 200025, China; ^2^Department of Ophthalmology, Ruijin Hospital, Luwan Branch, School of Medicine, The Jiaotong University, Shanghai 200025, China; ^3^Department of Ophthalmology, The Affiliated Hospital, School of Medicine, Ningbo University, Ningbo, Zhejiang 310000, China

## Abstract

*Purpose.* To determine the efficacy of intravitreal ranibizumab injection as adjuvant therapy in the treatment of neovascular glaucoma (NVG) accompanied by postvitrectomy diabetic vitreous hemorrhage (PDVH).* Methods.* Eighteen NVG patients (18 eyes) accompanied by PDVH were enrolled in this prospective, monocenter, 12-month, interventional case series. The consecutive 18 patients with an IOP ≥ 25 mmHg despite being treated with the maximum medical therapy were treated with intravitreal ranibizumab injections. Vitreous surgery or/with Ahmed valve implantation were indicated if no clinical improvement in vitreous haemorrhage and uncontrolled IOP was shown.* Results.* Ten patients got clear vitreous and controlled IOP only with 2.7 ± 1.8 injections of ranibizumab without additional surgery. Vitrectomy or/with Ahmed valve implantation was administered in the other 8 eyes due to uncontrolled VH and IOP. At follow-up month 12, all the 18 eyes gained clear vitreous. At month 12 BCVA improved significantly compared to baseline. The baseline and follow-up at month 12 IOP/medication usage were 36.7 ± 8.1 mmHg on 3.4 ± 0.7 medications and 16.2 ± 4.9 mmHg on 0.67 ± 0.77 medications, respectively.* Conclusions.* The findings suggest that intravitreal ranibizumab injection as adjuvant therapy for treatment of NVG accompanied by PDVH may be safe and potentially effective. This clinical trial is registered with NCT02647515.

## 1. Introduction

Postvitrectomy diabetic vitreous hemorrhage (PDVH) occurs frequently in patients who undergo pars plana vitrectomy for complications of proliferative diabetic retinopathy (PDR). These complications include vitreous hemorrhage, macula-involving or threatening tractional retinal detachment, rhegmatogenous retinal detachment, and combined traction and rhegmatogenous retinal detachment with or without hemorrhage [1–3]. It is reported by Brown et al. that the primary cause for reoperation in PDR was PDVH, with 14% of the rebleeding eyes regressing to no-light perception [4]. When PDVH occurs, the level of vascular endothelial growth factor (VEGF) becomes higher, which usually leads to neovascularization in the iris and the angle [5–8]. When neovascular fibrous tissues occupy the trabecular meshwork, aqueous outflow is disturbed, and NVG develops eventually. NVG aggravates retinal perfusion, leading to a self-enhancing cycle. A major reason of severe visual loss in these patients who received vitrectomy for PDR is due to NVG [9].

Based on the high failure rate associated with filtering surgery and valve implantation surgery, NVG is very difficult to deal with for glaucoma and retina specialists, especially accompanied by PDVH [8, 9]. Panretinal photocoagulation (PRP) is an effective way because it destroys large areas of retinal tissue and RPE. However, in advanced cases of NVG, PRP is difficult to perform because of turbid media due to increased IOP and vitreous bleeding. Cyclophotocoagulation is another alternative approach to lower IOP by reducing aqueous production from the ciliary body, rapidly improving retinal perfusion [10, 11]. Lowering IOP improves retinal perfusion but does not alter the underlying pathological mechanism that leads to neovascularization. Consequently, to decrease VEGF level is very critical in the treatment of NVG [12]. Lüke et al. demonstrated that intravitreal ranibizumab injection appears to be beneficial as an adjuvant treatment in NVG and rubeosis due to its antiangiogenic properties [9].

NVG is a potentially devastating glaucoma. Delayed diagnosis or poor management can cause complete loss of vision with intractable pain. In managing NVG, it is essential to treat both the elevated IOP and the underlying cause of the disease, such as PDVH. In view of the findings of the previous studies, we planned a prospective evaluation of the capacity of ranibizumab to treat the patients with NVG accompanied by PDVH.

## 2. Methods

The study was performed in the Department of Ophthalmology, Ruijin Hospital, Shanghai Jiaotong University School of Medicine, between January 2013 and December 2014. The research followed the tenets of the Declaration of Helsinki, and approval of the study was obtained from the institutional review board of Ruijin Hospital, Shanghai, China. All patients received a detailed explanation of the study and provided a written informed consent.

Inclusion criteria in this study were (i) age ≥ 18 years; (ii) PDVH obscuring the disc and vessels for more than 14 days and no evidence of retinal detachment after primary vitrectomy for PDR-related complications such as nonclearing VH, macula-involving or macula-threatening tractional retinal detachment, or fibrovascular proliferation with vitreoretinal adhesions; and (iii) NVG occurrence following PDVH less than 4 weeks (NVG was diagnosed when an IOP elevation of 22 mmHg or more was accompanied by neovascularization of the iris and/or the anterior chamber angle). The study exclusion criteria included (i) intraoperative use of long-acting gas or silicone oil in primary vitrectomy, repeat vitrectomy after primary vitrectomy for retinal diseases other than VH, and previous history of vitrectomy; (ii) previous intravitreal injection of ranibizumab or bevacizumab in either eye; (iii) previous intravitreal corticosteroids injection in either eye; (iv) pregnancy or current oral contraceptive intake; (v) usage of clopidogrel bisulfate or coumadin; (vi) uncontrolled hypertension and cardiac disease; and (vii) uncontrolled renal or liver disease.

The consecutive 18 patients were enrolled in this study and they were treated with intravitreal ranibizumab (0.5 mg in 0.05 mL) injection. Repeated intravitreal injection (0.5 mg in 0.05 mL) was given after 3 weeks in case of no obvious blood reabsorption while IOP was ≤25 mmHg and supplementary PRP was administered if needed and available by means of retina fluorescein angiography. Ranibizumab may have a considerably shorter half-life in the vitreous cavity in a vitrectomized eye than a nonvitrectomized eye. We chose 3 weeks as the interval between two ranibizumab injections based on the suggestion by Yeh et al. [13]. Vitrectomy with ranibizumab injection at the end of surgery was indicated if no clinical improvement in vitreous hemorrhage and IOP > 25 mmHg was noted 2 weeks after the initial ranibizumab injection. If recurrent VH occurred again in the revitrectomized eyes, repeated injection of ranibizumab would be undertaken again. For some cases, even though having clear vitreous, if IOP could not be controlled (≥30 mmHg) yet after vitreous surgery and ranibizumab injections as well as with medications, Ahmed valve implantation was administered.

All surgeries were performed by the same doctor (Xi Shen). Operations were performed with 23-gauge instruments (Alcon, USA). Procedures such as fibrovascular membrane dissection or endodiathermy were performed. Intraoperative bleeding was controlled by either endodiathermy or increasing the irrigation bottle height. In the surgery, new neovascularization in the retina was usually detected and supplementary PRP was performed further. Transient hypotony was made to identify any potential bleeding sites when operations were finished. At the end of surgery, balanced salt solution was used for globe filling. For some cases with uncontrolled IOP, Ahmed tube insertion was administered only or with vitrectomy at the same time. The Ahmed implantation procedure was as described previously [14].

All patients underwent a broad ophthalmologic examination at baseline and postoperative week 2 through month 12. At each visit, patients were assessed for logMAR best-corrected visual acuity examination (BCVA) (light perception vision was assigned as logMAR vision of 2.6, hand motion vision as logMAR vision of 2.3, and counting finger vision as logMAR vision of 1.85 [15]), recurrent VH, number of ranibizumab injections, IOP (Goldmann applanation tonometry), number of glaucoma medications (topical and oral), and surgical interventions.

The criteria for success at 12 months postoperatively were IOP ≤ 21 mmHg without the necessity for adjunctive medications and IOP ≤ 18 mmHg with 1 adjunctive medication and at the same time the vitreous cavity is clear.

Statistical analysis was performed with SPSS software version 17.0 (IBM, Inc., USA) using Student's *t*-test, with a *P* value < 0.05 considered statistically significant.

## 3. Results

### 3.1. Baseline Characteristics

Between October 2012 and September 2014, 18 eyes of 18 patients were enrolled in the study. Baseline patient demographic data are summarized in Table 1. The patients ranged in age from 27 to 73 (average: 51.1 ± 14.8) in the study. Most cases (15/18) had insulin-dependent diabetic mellitus. PDVH occurred between 4 and 36 weeks (average: 15.2 ± 9.7 weeks) after primary vitrectomy and NVG generated immediately between 1 and 4 weeks (average: 2.2 ± 0.9 weeks) after PDVH. No patient discontinued in the study during the complete 12-month follow-up (Table 1).

### 3.2. Recurrent VH and Treatments

In our study, 18 consecutive patients were enrolled. After the first injection of ranibizumab, six eyes got clear vitreous from two weeks to 8 weeks (average: 5.3 ± 2.4 weeks) with an average of 1.5 ± 0.5 injections, and no recurrent VH occurred during follow-up. Although another 4 eyes (cases 1, 4, 13, and 16) also obtained clear vitreous following first injection, second- or third-episode recurrent VH was noted during remaining follow-up. After an average of 16.0 ± 3.5 weeks and 4.3 ± 0.9 injections, the four eyes finally regained vitreous clear-up. Total reabsorption time of recurrent VH was 9.6 ± 6.1 weeks (range: 2–27 weeks) in the ten eyes and their IOP was controlled ≤21 mmHg with or without antiglaucoma medications. Due to clear vitreous and controlled IOP in the ten eyes, no surgery was needed during follow-up. Based on uncontrolled VH and IOP >25 mmHg in another 8 eyes after the initial ranibizumab injection, vitreous surgery with supplementation of retinal photocoagulation was administered and at the end of surgery ranibizumab was reinjected intravitreally. After this kind of treatment, four cases (cases 7, 8, 12, and 15) still received mean 3.5 ± 0.58 ranibizumab injections because of uncontrolled recurrent VH; case 14 received another 3 ranibizumab injections due to uncontrolled NVG. The five eyes gained clear vitreous and controlled IOP at 12-month follow-up with or without antiglaucoma medications. For cases 10, 17, and 18, due to recurrent VH and/or uncontrolled IOP, the remaining three of the 8 eyes received repeated injections of ranibizumab and revitrectomy; two of the 3 eyes (cases 17 and 18) received Ahmed valve implantation due to uncontrolled IOP. Finally, all the 18 eyes got clear vitreous at the follow-up month 12 and the total reabsorption time was 17.83 ± 12.71 weeks (Table 2).

### 3.3. Intraocular Pressure (IOP) and Antiglaucomatous Medication/Surgery

**Figure 1:**
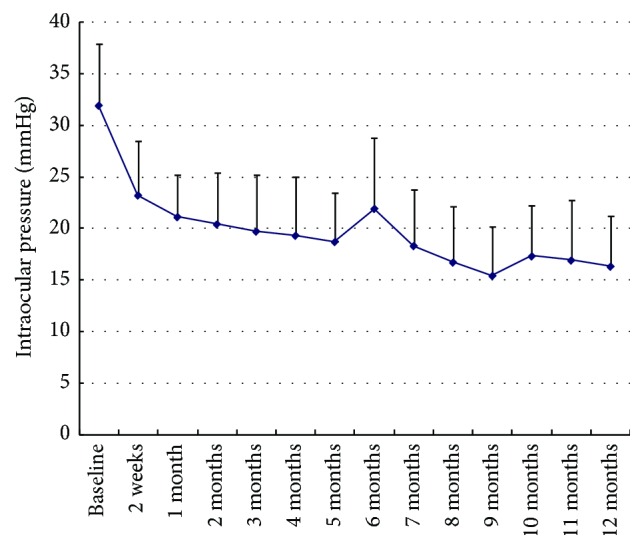
Intraocular pressure revealed a significant reduction compared to baseline at every follow-up visit during the 12 months (*P* < 0.01).

The mean IOP was 36.7 ± 8.1 mmHg at baseline. At the first follow-up, the baseline IOP decreased significantly, ranging between 15 and 30 mmHg (23.2 ± 5.3 mmHg) in all the 18 eyes. During the 12-month follow-up, after additional treatments with vitrectomy or/with Ahmed valve implantation and medications usage, at the remaining visits (week 2 till month 12), the mean IOP value was under 23.2 ± 5.3 mmHg. At follow-up month 12, the mean IOP was 16.2 ± 4.9 mmHg (Figure 1).

**Figure 2:**
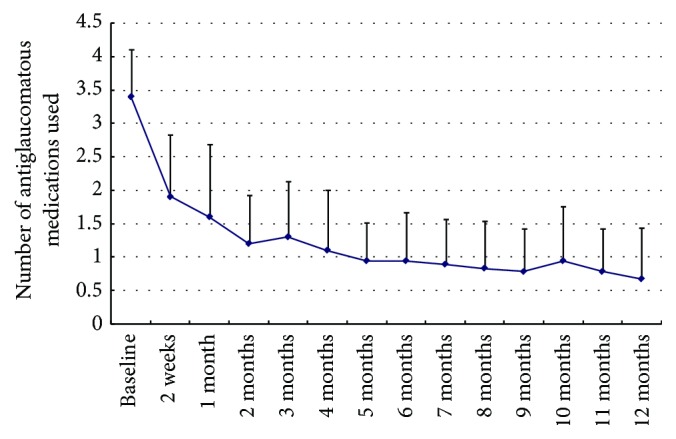
Graph showing changes in the number of antiglaucomatous medications applied from baseline to 12 months after treatment. At every follow-up visit, compared to baseline, the number of antiglaucomatous medications undertaken significantly reduced (*P* < 0.01).

At baseline, on average, 3.4 ± 0.70 topical antiglaucomatous medications were applied, and ten patients also received oral acetazolamide. At every follow-up visit, compared to baseline, the number of antiglaucomatous medications applied significantly reduced (*P* < 0.01). After month 6, the number of antiglaucomatous medications applied was between 0 and 2 at the following visits, and no patients received oral acetazolamide. At 12-month follow-up, only 0.67 ± 0.77 antiglaucomatous medications were applied (Figure 2).

There were significant differences in IOP and antiglaucomatous medications usage between baseline and each visit during 12-month follow-up. At follow-up month 12, success was achieved in 83.3% of the 18 subjects.

### 3.4. Best-Corrected Visual Acuity (BCVA, logMAR)

**Figure 3:**
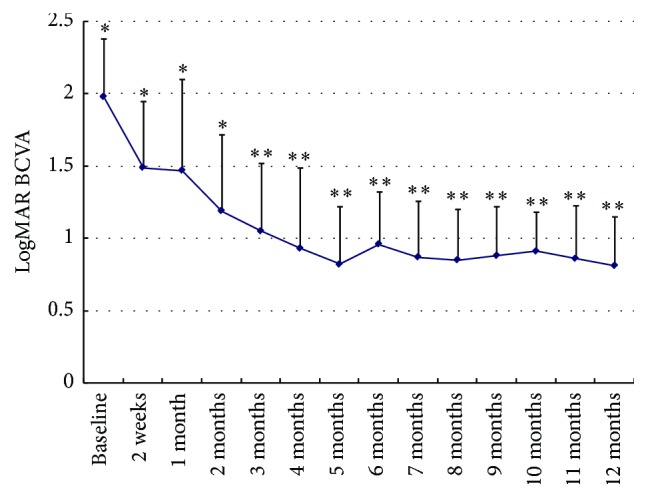
Graph showing changes in mean logarithm of the minimum angle of resolution (logMAR) best-corrected visual acuity (BCVA) from baseline to 12 months after treatment. At earlier visits follow-up (week 2 till month 2), BCVA revealed a modest improvement compared to baseline (*P* < 0.05). At the remaining visits (month 3 till month 12), the improvement of BCVA reached the significant level compared to baseline (*P* < 0.01). *∗* represents *P* < 0.05; *∗∗* represents *P* < 0.01.

At baseline, the mean BCVA was 1.99 ± 0.40. At earlier visits follow-up (week 2 till month 2), BCVA revealed a modest improvement compared to baseline (*P* < 0.05). At the remaining visits (month 3 till month 12), the improvement of BCVA reached the significant level (*P* < 0.01). At month 12, BCVA was 0.81 ± 0.34 (*P* = 0.000, Figure 3).

### 3.5. Adverse Events

No unmanageable intraoperative or postoperative complications developed in this series, such as endophthalmitis, retinal detachment, suprachoroidal hemorrhage, cellulitis, phthisis bulbi, or persistent hypotony [IOP <5 mmHg]. As for systemic adverse events such as myocardial infarction or cerebrovascular accidents, there were no occurrences in any of the patients in this study.

## 4. Discussion 

This prospective, controlled interventional case series reports for the first time on an effective IOP control and reabsorption of recurrent hemorrhage after adjuvant intraocular ranibizumab injection in addition to appropriate antiglaucomatous treatment and therapy of the NVG accompanied with PDVH lasting for the complete 12-month follow-up.

Recurrent VH after successful diabetic vitrectomy is not uncommon, although adequate retinal ablation plus sclerotomy site cryotherapy has been undertaken. Previous reports showed that in the early postoperative period, injured vessels, residual fibrovascular tissue, or early regrowth of neovascularization may be the causes of bleeding [16]; late recurrent hemorrhage is more likely associated with neovascularization from the peripheral retina or instrument entry sites [17]. Many methods have been used to treat postvitrectomy persistent or recurrent hemorrhage, including gas-fluid exchange, panretinal cryotherapy, vitreous lavage, and dissection of entry-site fibrovascular proliferation [17, 18]; however further recurrent hemorrhage occurred frequently.

When PDVH occurs and persists, NVG usually appears immediately, which may be due to the VEGF, diffusing throughout the globe and leading to neovascularization. It was reported that high level of VEGF induced PDVH and NVG in patients with PDR who received vitrectomy [12]. NVG aggravates retinal perfusion, leading to a self-enhancing cycle. NVG after vitrectomy for PDR is the most serious complication and portends very poor prognosis. As long as only NVG with PDVH occurs, the treatments become very difficult to be administered [5–9].

Bevacizumab (Avastin, Roche), a recombinant monoclonal antibody that binds to all subtypes of vascular endothelial growth factor (VEGF), has been shown to induce effective regression of retinal neovascularization secondary to proliferative diabetic retinopathy (PDR) [6–8]. Recently, numerous reports have focused on the adjunctive use of bevacizumab to reduce postoperative VH in vitrectomy for PDR [19–22]. However, in China, intravitreal bevacizumab injection is considered as an off-label treatment in DR and PDR. Ranibizumab (Lucentis, Novartis), another anti-VEGF drug, a fragment of a recombinant humanized IgG1 monoclonal antibody that inhibits all isoforms of the human VEGF-A, has been shown to be beneficial as an adjuvant treatment in neovascular glaucoma and rubeosis due to its antiangiogenic properties [9]. A current study by Li et al. reported that ranibizumab plus combined surgery for treatment of NVG with VH was effective [23]. Based on the previous findings, we hypothesize that intravitreal injection of ranibizumab in eyes with NVG companied with PDVH may induce regression of new vessels and reduce the possibility of repeated bleeding, thus shortening the reabsorption time, lowering higher IOP, and reducing the need for surgery.

Eighteen consecutive patients were enrolled in our study. In the present study, 10 patients (10 eyes) obtained clear vitreous and controlled IOP at the end of complete 12-month follow-up only by ranibizumab injection (2.7 ± 1.8 injections) with or without supplementary PRP, although second or third episode recurrent VH was noted in some cases. For the remaining 8 eyes in this study, in order to decrease the impact of uncontrolled VH and higher IOP on retina, vitreous surgery was administered. During follow-up, reinjection of ranibizumab was administered again due to recurrent VH occurred and uncontrolled NVG. After this kind of treatment, five of the 8 eyes gained clear vitreous and controlled IOP finally at 12-month follow-up. The remaining three of the 8 eyes received vitrectomy or/with Ahmed valve implantation because of uncontrolled recurrent VH and/or uncontrolled IOP during follow-up. Yeh et al. reported that in their study after treatment of intravitreal bevacizumab injection for recurrent VH after diabetic vitrectomy, no surgery was needed [13]. However, in our study, in 8 eyes (44.4%), revitrectomy and/or valve implantation was still needed to be performed. We considered that this result was due to the fact that our patients encountered NVG and PDVH at the same time. Although effects of ranibizumab injection are known to be transient, especially in vitrectomized eyes, to some degree, the ranibizumab still can inhibit the effect of VEGF, resulting in improving absorption of VH and inducing regression of angle and retinal neovascularization, lowering higher IOP consequently. However, due to the difficulty of treatment for NVG and PDVH, further surgical procedures, including vitrectomy or valve implantation, cannot be avoided completely.

Intraocular pressure following comprehensive treatment was one of the major criteria of therapeutic success. NVG usually resulted from anterior chamber angle obstruction due to angle neovascularization and peripheral anterior synechiae. Before complete formation of synechiae, IOP may be controlled without further need for surgical procedures by treatment with ranibizumab injection [9]. Lüke et al. showed if neovascularizations of the anterior chamber angle were present, ranibizumab alone in combination with adequate PRP has been demonstrated to be sufficient to achieve a permanent regression of angle involvement, to prevent synechiae, and to reduce the intraocular pressure rapidly [9]. In our study, the rapid regression of iris rubeosis and absorption of VH was combined with a significant IOP reduction in major cases after 14 days. At the first follow-up, the baseline IOP (36.7 ± 8.1 mmHg) decreased significantly, ranging between 15 and 30 mmHg (23.2 ± 5.3 mmHg) in all the 18 eyes. The acute IOP reduction in those cases might be due to nonsynechial angle closure. After treatment with ranibizumab injection, the regression of neovascularization and inflammation allowed improved drainage via the noninvolved trabecular meshwork. During follow-up, repetatus elevated IOP and recurrent VH might cause irreversible synechiae formation, resulting in no or lower response to anti-VEGF treatment. Therefore, intravitreal anti-VEGF treatment alone seems to be insufficient in those pathologically advanced stages which require an additional treatment of the ischemic retina, such as vitrectomy or/with valve implantation. After adjuvant ranibizumab injection, a significant reduction of antiglaucomatous medication was also observed in the study at each visit compared to baseline, especially after 6-month follow-up, the number of antiglaucomatous medications applied was between 0 and 2 at the remaining following visits, and no patients received oral acetazolamide.

In addition to the absorption of recurrent vitreous hemorrhage and the IOP control, a significant improvement of BCVA was evident in our study. Due to more episodes of recurrent bleeding in early stage, BCVA revealed a modest improvement compared to baseline (*P* < 0.05) at earlier visits follow-up (week 1 till month 3). At the remaining visits (month 4 till month 12), with the IOP control and clearance of vitreous cavity improvement, BCVA reached the significant level (*P* < 0.01). The increase of BCVA was due to the well-graduated combined treatment regime, including reinjection of ranibizumab, vitrectomy, and/or valve implantation, which resulted in regression of corneal and macular oedema, clearance of vitreous, and IOP control.

In summary, our study suggests that repeated intravitreal ranibizumab injection may be effective as adjuvant therapy in the treatment of PDVH accompanied by NVG in facilitating reabsorption of vitreous blood, decreasing higher IOP, and reducing additional vitreous surgeries. However, this study has certain limitations. First, the study was designed without control group, just to compare the characteristics between baseline and posttreatment. Second, the study only had a small number of cases. Although there were no serious side effects found in this study with repeated intravitreal ranibizumab injection, further study with prospective design and a larger case number may be necessary to confirm the efficacy and safety of the treatment.

## Figures and Tables

**Table 1:** Baseline patient characteristics.

Patient	Eye	Gender	Age (year)	Lens status	LogMAR BCVA	IOP (mmHg)	No. of antiglaucomatous medications used	PDVH after vitrectomy (weeks)	NVG after PDVH (weeks)
1	Right	Female	45	Pseudophakic	1.6	38	4	9	2
2	Left	Male	27	Phakic	1.4	26	2	4	3
3	Right	Male	54	Pseudophakic	1.6	41	3	12	1
4	Right	Male	64	Pseudophakic	2.6	51	4	22	2
5	Right	Female	32	Phakic	1.85	29	3	16	3
6	Right	Male	59	Pseudophakic	2.3	33	4	8	4
7	Left	Male	69	Pseudophakic	1.85	30	3	7	1
8	Left	Female	73	Pseudophakic	1.85	35	3	28	2
9	Right	Male	71	Pseudophakic	1.85	35	4	36	2
10	Right	Female	46	Pseudophakic	1.6	42	4	25	2
11	Left	Male	39	Pseudophakic	2.3	36	4	9	3
12	Right	Male	33	Phakic	1.5	33	3	5	4
13	Left	Male	42	Pseudophakic	1.85	47	3	16	3
14	Right	Male	66	Pseudophakic	1.85	25	2	29	2
15	Left	Male	58	Pseudophakic	2.3	35	3	23	1
16	Left	Female	44	Pseudophakic	2.6	42	4	9	1
17	Right	Female	38	Pseudophakic	2.3	40	4	5	2
18	Right	Female	62	Pseudophakic	2.6	43	4	11	2

LogMAR = logarithm of minimum angle of resolution; BCVA = best-corrected visual acuity; IOP = intraocular pressure; no. of antiglaucomatous medications used = number of antiglaucomatous medications used; PDVH = postvitrectomy diabetic vitreous hemorrhage; NVG = neovascular glaucoma.

**Table 2:** Clinical characteristics of recurrent VH and treatments.

Patients	Number of rebleeding episodes	Number of IVIR for RVH	No. of S.PRP	TRT of RVH (weeks)	Surgical intervention (V/AVI)
1	2	3	1	13	No
2	1	2	1	8	No
3	1	2	1	7	No
4	2	5	2	15	No
5	1	1	0	5	No
6	1	2	1	7	No
7^*∗*^	2	5	/	29	V
8^*∗*^	3	6	/	38	V
9	1	1	0	2	No
10^*∗*^	2	4	/	21	V/V
11	1	1	1	3	No
12^*∗*^	2	6	/	29	V
13	2	4	1	15	No
14^*∗*^	1	5	/	11	V
15^*∗*^	3	5	/	17	V
16	3	6	1	21	No
17^*∗*^	1	4	/	36	V/V/AVI
18^*∗*^	2	5	/	44	V/V + AVI

No. of S.PRP = number of supplementary PRP in patients without surgical intervention; IVIR = intravitreal injection of ranibizumab; RVH = recurrent vitreous hemorrhage; TRT = total reabsorption time; V = vitrectomy; AVI = Ahmed valve implantation; V/V = two vitrectomies at different time; *∗* represents patients having surgical intervention.
